# Differential expression profile of plasma exosomal microRNAs in acute type A aortic dissection with acute lung injury

**DOI:** 10.1038/s41598-022-15859-3

**Published:** 2022-07-08

**Authors:** Chiyuan Zhang, Hui Bai, Lei Zhang, Yanfeng Zhang, Xuliang Chen, Ruizheng Shi, Guogang Zhang, Qian Xu, Guoqiang Lin

**Affiliations:** 1 Department of Cardiovascular Surgery, Xiangya Hospital, Central South University, Xiangya Rd 87, Changsha, 410008 Hunan China; 2 Department of Cardiovascular Medicine, Xiangya Hospital, Central South University, Xiangya Rd 87, Changsha, Hunan China; 3 Present Address: Department of Cardiovascular Medicine, The Third Xiangya Hospital, Central South University, Tongzipo Rd 138, Changsha, Hunan China

**Keywords:** Cardiovascular biology, Predictive markers, Cardiovascular diseases, Respiratory tract diseases

## Abstract

MicroRNAs (miRNAs) packaged into exosomes mediate cell communication and contribute to the pathogenesis of acute type A aortic dissection (ATAAD) with acute lung injury (ALI). The expression profile of plasma exosomal miRNAs in ATAAD patients with ALI hasn’t been identified. We performed a miRNA-sequencing to analyze the differentially expressed miRNAs (DE-miRNAs) of circulating exosomes in ATAAD patients with ALI compared to patients without ALI, founding 283 specific miRNAs in two groups. We respectively selected the top 10 downregulated and upregulated DE-miRNAs for further studies. The predicted transcription factors (TFs) of these DE-miRNAs were SMAD2, SRSF1, USF1, etc. The Gene Ontology (GO) and Kyoto Encyclopedia Genes and Genomes (KEGG) analysis predicted their target genes mainly involved acute inflammatory response, cell junction, cytoskeleton, NF-κB signaling pathway, etc. Construction and analysis of the PPI network revealed that RHOA and INSR were considered hub genes with the highest connectivity degrees. Moreover, we confirmed two exosomal miRNAs (hsa-miR-485-5p and hsa-miR-206) by real-time quantitative polymerase chain reaction (RT-qPCR) in a validation cohort. Our study identified a plasma exosomal miRNAs signature related to ATAAD with ALI. Certain DE-miRNAs may contribute to the progression of this disease, which help us better understand the pathogenesis of ATAAD with ALI.

## Introduction

Acute Stanford type A aortic dissection (ATAAD) is a life-threatening cardiovascular disease with urgent onset, rapid progression and high mortality, which often requires emergency surgical treatment^1^. Acute lung injury (ALI) is one of its preoperative complications that seriously affect the surgical outcome^2^. In recent years, studies have shown that about 50% of ATAAD patients are complicated with ALI before surgery^2^. This not only lengthens the duration of postoperative intubation, mechanical ventilation and intensive care unit (ICU) stay but also leads to a death rate of about 25–67%, greatly increasing the perioperative mortality and seriously affecting the prognosis of ATAAD patients^3^.

Inflammation is involved in the occurrence and development of ATAAD with ALI. The progression of local vascular inflammation caused by intima tear and false lumen formation in aortic dissection can develop into systemic inflammation and result in multiple organ damage including lung^4,5^. Some studies indicated that inflammatory reactants such as interleukin-6 (IL-6), C-reactive protein (CRP) and monocyte chemoattractant protein-1 (MCP-1) were significantly increased in blood or lung tissues from ATAAD patients with ALI, and these reactants could directly or indirectly promote the apoptosis and barrier dysfunction of pulmonary microvascular endothelial cells (PMVECs), which contributed to the elevation of endothelial permeability and the formation of ALI^6–8^. However, the underlying mechanism has not been well elucidated.

Exosomes are microvesicles (30–100 nm in diameter) that originated in the endosomal membrane compartment and formed in the cytoplasm^9^. They are secreted by almost all living cells and exist in various body fluids such as plasma, urine and synovial fluid^10^. Exosomes mediate cell-to-cell communication by transporting their cargo including proteins, lipids, mRNAs and RNAs to target cells^10,11^. microRNAs (miRNAs) account for 41.72% of all RNAs in exosomes^12^ and they function as posttranscriptional regulators of gene expression by interacting with target mRNAs, playing a critical role in maintenance tissues and organ homeostasis^13–15^. For instance, miR-208a was shown to directly increase β-myosin heavy chain (MHC), which was associated with arrhythmia, fibrosis and hypertrophic growth^14^. miR-21and miR-16 were reported to impair barrier integrity and regulating inflammatory responses through downregulating Ras homolog gene family member B (RhoB) and interleukin-6 (IL-6), which were involved in intestinal and pulmonary mucosa, such as ALI or inflammatory bowel disease (IBD)^15^. Besides, increasing evidence has demonstrated that the potential role of exosomal miRNAs can positively affect the progression of cardiovascular diseases such as acute coronary syndrome, heart failure, pulmonary arterial hypertension, rheumatic valvular disease and aortic disease^16^. However, little research has been conducted on their influence on ATAAD with ALI. Research into plasma exosomal miRNAs may bring us a new perspective as we consider the underlying mechanism of the multiple organ disorder (e.g. lung) in ATAAD. Thus, we investigated plasma exosomal miRNAs profiles in ATAAD patients with ALI in order to partly explain the disease’s pathophysiology.

## Results

### Description of study patients

**Figure 1:**
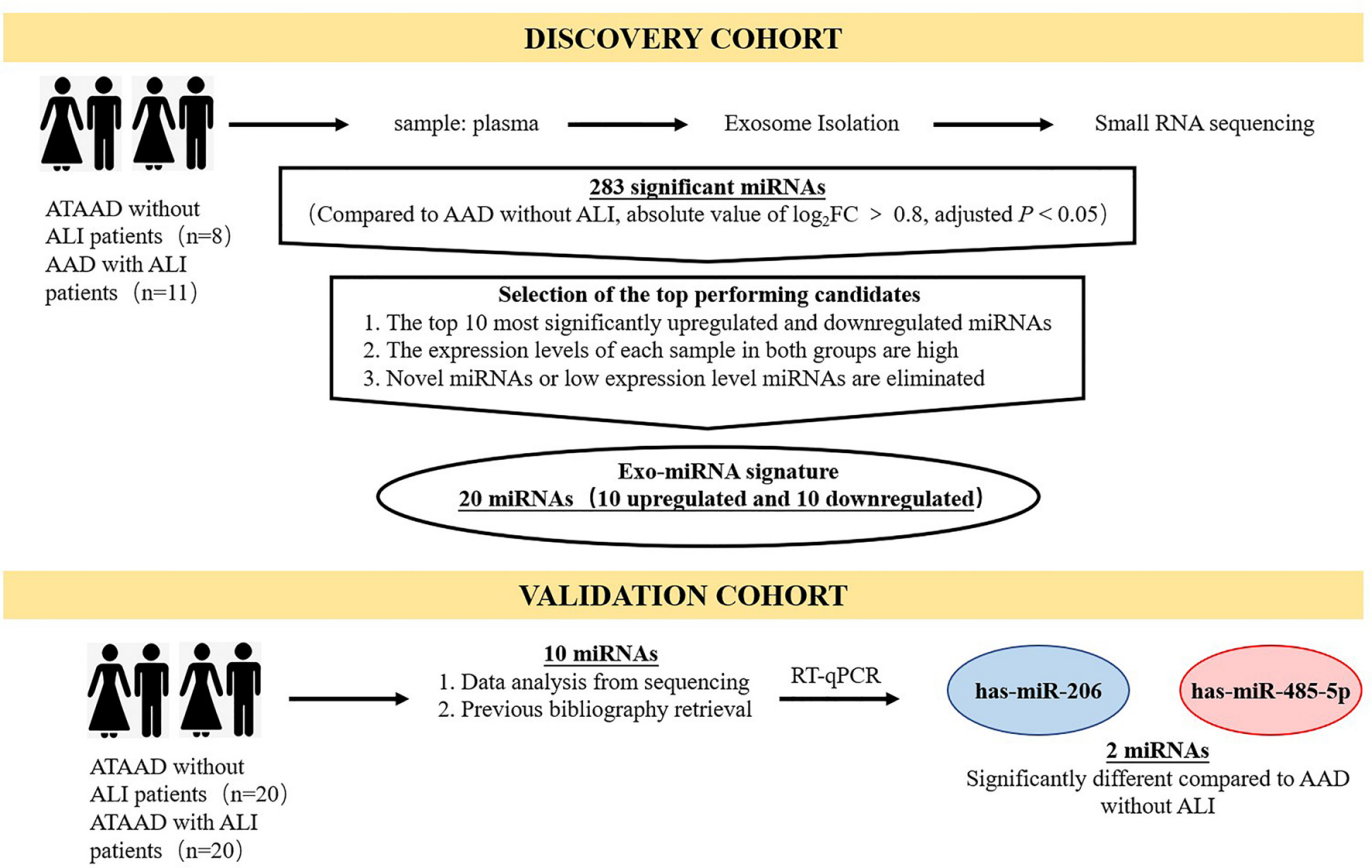
Schematic overview of the strategy for identifying an plasma exosomal miRNAs signature associated with ATAAD with ALI. Our study design involved a discovery phase including ATAAD (n = 19) with ALI or without ALI by small RNA sequencing. In a second validation phase with a large ATAAD cohort (n = 40), including with ALI or without ALI, 2 miRNAs were finally validated by RT-qPCR. *ATAAD* acute type A aortic dissection; *ALI* acute lung injury, *RT-qPCR* real-time quantitative polymerase chain reaction, *miRNAs* microRNAs.

This is a case–control study that included ATAAD patients with and without ALI as a discovery cohort and validation cohort (Fig. 1). We recruited 19 ATAAD patients treated as the discovery cohort for plasma exosomal miRNAs sequencing, and they were categorized ATAAD with ALI (ALI group, n = 11) and ATAAD without ALI (Non-ALI group, n = 8). Additional 20 ATAAD patients were enrolled for each group as the validation cohort. The clinical and demographic characteristics of study patients in the discovery and validation cohort were presented in Supplementary Table S1 and S2 online. There is no significant difference in most clinical parameters between the non-ALI group and the ALI group. In the discovery cohort, patients in the ALI group had a higher respiratory and heart rate and lower arterial oxygen tension (P_aO2_)/inspiratory oxygen fraction (F_iO2_) and P_aO2_. Additionally, left ventricular ejection fraction (LVEF) was statistically different between the two groups, but not clinically significant. In the validation cohort, the value of P_aO2_ and P_aO2_/F_iO2_ were lower in patients in the ALI group, and these patients had more hypertension.

### Isolated plasma exosomes characterization

Exosomes from plasma were identified by their morphology, diameter distribution and enriched exosome markers such as CD63 and TSG101. The transmission electron microscopy (TEM) image (Supplementary Fig. S1a online) showed that the isolated exosomes were spherical structures with a diameter of about 30–100 nm. Nanoparticle tracking analysis (NTA) (Supplementary Fig. S1b online) revealed that the extracted exosomes had a median diameter of 58.9 nm and their concentration was 4.1 × 10^9^/mL. Besides, these exosomes were positive for enriched exosome markers CD63 and TSG101 and negative for Calnexin (an intracellular compartment marker that was absent in exosomes) on Western blot (Supplementary Fig. S1c online). The results above indicated that the plasma exosomes had been adequately purified.

### Exosomal miRNAs signature in ATAAD with ALI

**Figure 2:**
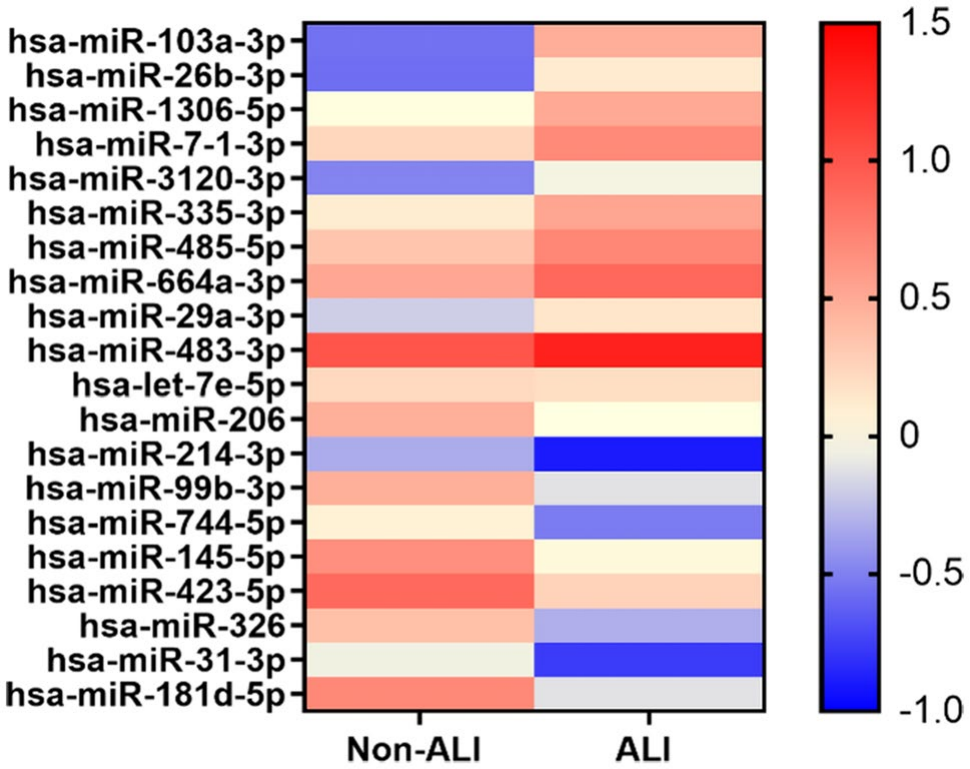
Heat map of the top 10 downregulated and upregulated DE-miRNAs in Non-ALI group vs ALI group. The color key indicates the expression level of the miRNAs. *DE-miRNAs* differentially expressed miRNAs; *Non-ALI group* patients with acute type A aortic dissection without acute lung injury, *ALI group* patients with acute type A aortic dissection with acute lung injury.

To identify the plasma exosomal miRNAs profile of ATAAD patients with ALI, we used the small RNA sequencing method. As shown in Supplementary Fig. S2 online, we found that 283 exosome-specific miRNAs (163 downregulated and 120 upregulated) were significantly different (Non-ALI group vs ALI group; Cut-off: adjusted *p* < 0.05, │fold change│ > 1.8). Then, we eliminated those novel miRNAs or miRNAs with low fragments perkiobase million (FPKM) in each sample, and the top 10 downregulated (hsa-miR-181d-5p, hsa-miR-31-3p, hsa-miR-326, hsa-miR-423-5p, hsa-miR-145-5p, hsa-miR-744-5p, hsa-miR-99b-3p, hsa-miR-214-3p, hsa-miR-206, hsa-let-7e-5p) and upregulated (hsa-miR-103a-3p, hsa-miR-26b-3p, hsa-miR-1306-5p, hsa-miR-7-1-3p, hsa-miR-3120-3p, hsa-miR-335-3p, hsa-miR-485-5p, hsa-miR-664a-3p, hsa-miR-29a-3p, hsa-miR-483-3p) miRNAs were shown in Table 1 and Fig. 2.

**Table 1:** The top 10 significantly upregulated and downregulated miRNAs.

Downregulated miRNAs		Upregulated miRNAs	
miRNA	log_2_FC	miRNA	log_2_FC
hsa-miR-181d-5p	−2.74	hsa-miR-103a-3p	3.45
hsa-miR-31-3p	−2.33	hsa-miR-26b-3p	2.29
hsa-miR-326	−2.30	hsa-miR-1306-5p	1.67
hsa-miR-423-5p	−2.06	hsa-miR-7-1-3p	1.50
hsa-miR-145-5p	−2.05	hsa-miR-3120-3p	1.47
hsa-miR-744-5p	−2.01	hsa-miR-335-3p	1.38
hsa-miR-99b-3p	−1.97	hsa-miR-485-5p	1.21
hsa-miR-214-3p	−1.89	hsa-miR-664a-3p	1.21
hsa-miR-206	−1.58	hsa-miR-29a-3p	1.17
hsa-let-7e-5p	−0.97	hsa-miR-483-3p	0.97

*FC* fold change, *miRNAs* microRNAs.

### Prediction of transcription factors (TFs) of differentially expressed miRNAs (DE-miRNAs)

**Figure 3:**
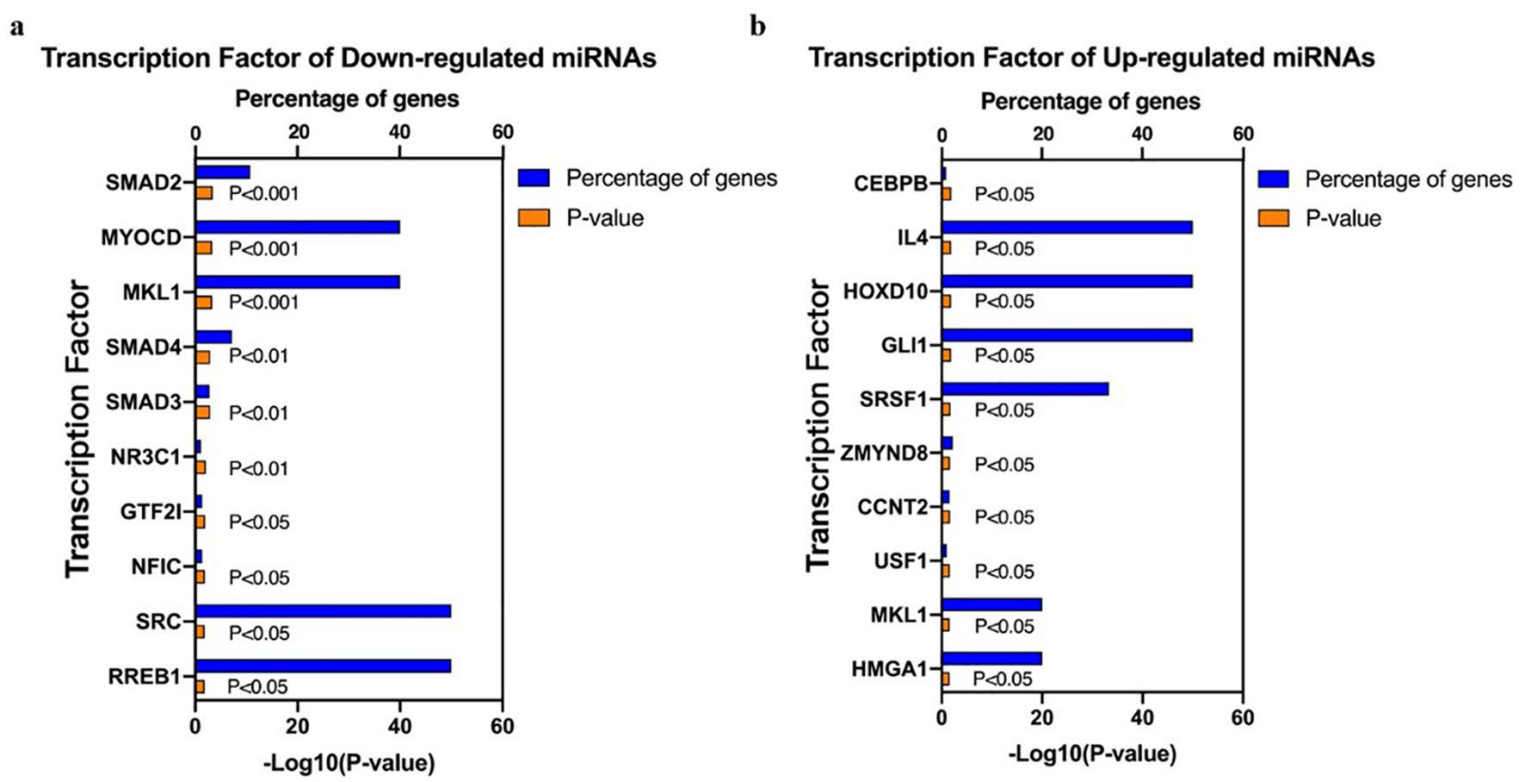
The top 10 predictive TFs of DE-miRNAs. (**a**) The top 10 TFs of downregulated DE-miRNAs; (**b**) the top 10 TFs of upregulated DE-miRNAs. *TFs* transcription factors; *DE-miRNAs* differentially expressed miRNAs.

Based on the sequencing results above, we concentrated on the following functional study of the top 10 candidates down- and upregulated miRNAs. Their upstream TFs were predicted by FunRich software. We listed the top 10 TFs for these DE-miRNAs, which were shown in Fig. 3a,b. For downregulated DE-miRNAs, the top 10 TFs were SMAD2, MYOCD, MKL1, SMAD4, SMAD3, NR3C1, GTF2I, NFIC, SRC and RREB1. For upregulated DE-miRNAs, the top 10 TFs were CEBPB, IL4, HOXD10, GLI1, SRSF1, ZMYND8, CCNT2, USF1, MKL1 and HMCA1.

### Gene Ontology (GO) functional and Kyoto Encyclopedia Genes and Genomes (KEGG) pathway analysis

**Figure 4:**
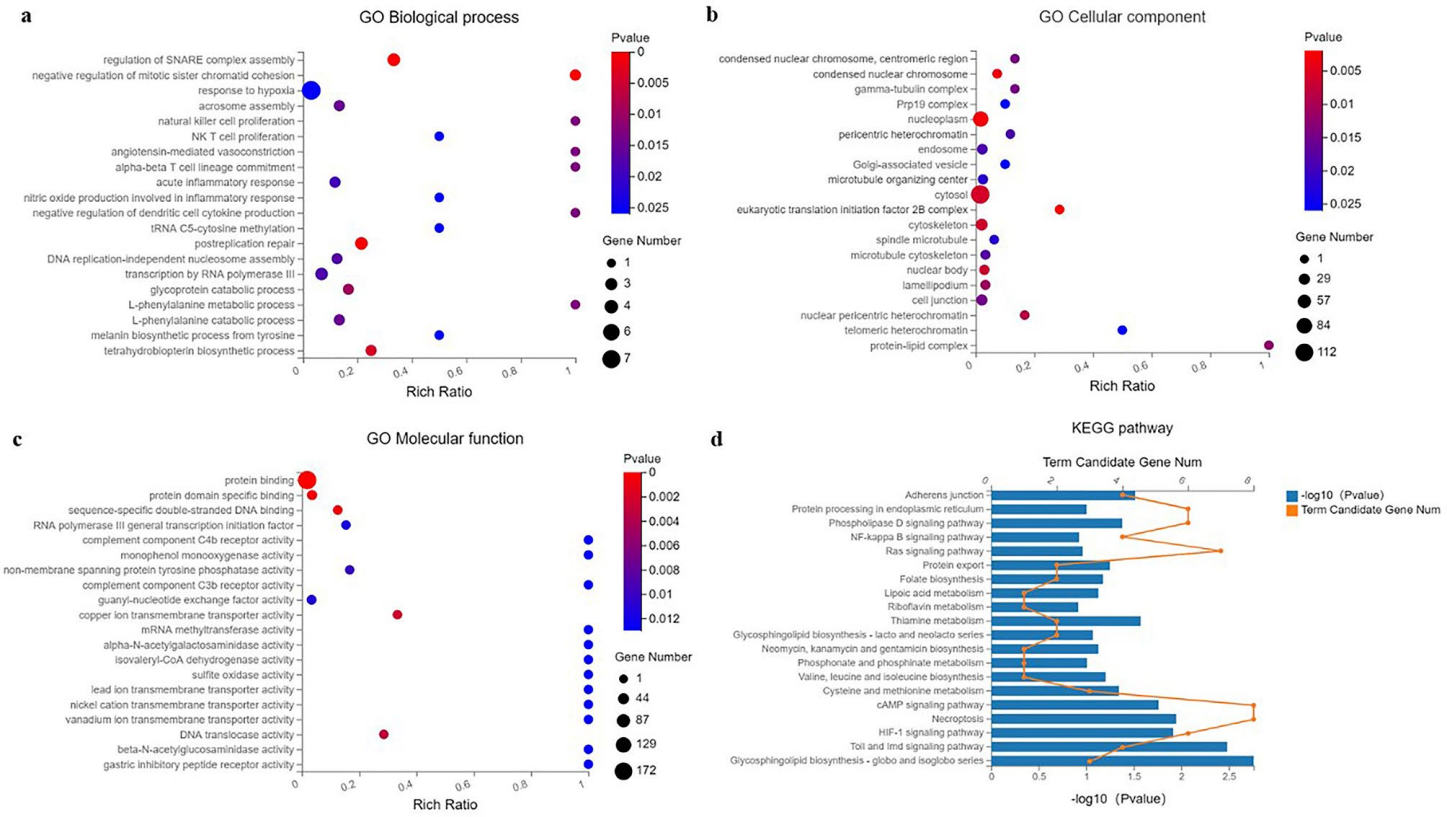
GO and KEGG pathway analysis for the target genes of the top 10 DE-miRNAs. (**A–C**) The top 20 associated biological processes (**a**), cellular components (**b**) and molecular functions (**c**) in which the target genes were significantly enriched; (**d**) the top 20 related KEGG pathways of target genes. *GO* Gene Ontology, *KEGG* Kyoto Encyclopedia of Genes and Genomes.

Initially, we used the TargetScan, miRbase and RNAhybrid websites to predict the target genes of the top 10 DE-miRNAs. Additionally, to better understand the biological function and pathways of the predictive target genes, we performed GO and KEGG pathway analysis. The biological process (BP), cellular component (CC) and molecular functions (MF) of GO analysis were summarized in Fig. 4a–c. The changes of BP were enriched in the response to hypoxia, natural killer (NK) cell proliferation, NK T cell proliferation, acute inflammatory response, etc. (as shown in Fig. 4a), and the changes of CC were enriched in the cell junction, cytoskeleton, etc. (as shown in Fig. 4b). Figure 4c suggested that the changes of MF were manifested in the protein binding, complement component C4b and C3b receptor activity, sulfite oxidase activity, etc. The KEGG pathway analysis was presented in Fig. 4d, and these predicted target genes were primarily involved in adhesion junction, necroptosis, NF-κB signaling pathway, etc.

### Protein–protein interaction (PPI) network construction

**Figure 5:**
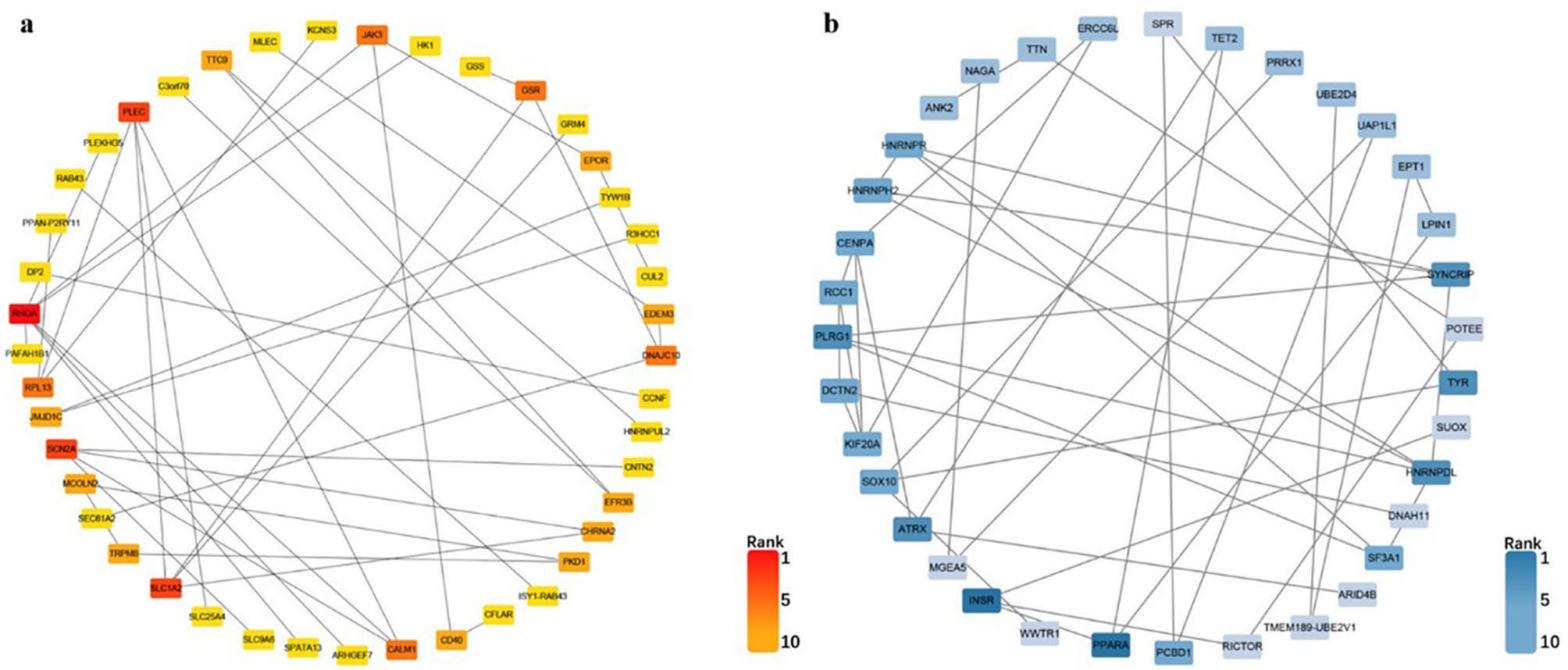
The PPI network of the predictive target genes of the top 10 DE-miRNAs. (**a**) The red rectangle represents the predicted upregulated gene of downregulated DE-miRNAs; (**b**) the bule rectangle represents the predicted downregulated gene of upregulated DE-miRNAs. The more node pairs, the darked the color is, which indicates the higher the rank of hub gene is. *PPI* protein–protein interaction, *DE-miRNAs* differentially expressed miRNAs.

We submitted those predictive target genes of the candidate DE-miRNAs to the STRING database and constructed PPI networks for them, which was shown in Fig. 5a,b. Using a comprehensive score greater than 0.4, we obtained 55 and 125 node pairs for target genes of downregulated and upregulated DE-miRNAs, respectively. Then, to identify the hub genes of the two PPI networks, we input these node pairs into Cytoscape, and the top 10 hub genes calculated by degree scoring were presented in Table 2. In the target genes of downregulated DE-miRNAs, the hub genes were RHOA, SLC1A2, SCN2A, PLEC, JAK3, GSR, CALM1, RPL13, DNAJC10 and EFR3B; in the target genes of upregulated DE-miRNAs, the hub genes were INSR, PPARA, HNRNPDL, TYR, ATRX, SYNCRIP, PLRG1, HNRNPH2, DCTN2 and PCBD1.

**Table 2:** The top 10 hub genes in the PPI networks.

Upregulated candidate genes		Downregulated candidate genes	
Name	Nodes	Name	Nodes
RHOA	7	INSR	6
SLC1A2	4	PPARA	6
SCN2A	4	HNRNPDL	5
PLEC	4	TYR	5
JAK3	3	ATRX	5
GSR	3	SYNCRIP	5
CALM1	3	PLRG1	5
RPL13	3	HNRNPH2	4
DNAJC10	3	DCTN2	4
EFR3B	2	PCBD1	4

*PPI* protein–protein interaction.

### Validation of DE-miRNAs by real-time quantitative polymerase chain reaction (RT-qPCR)

To confirm the disorder of specific plasma exosomes miRNAs in response to ATAAD with ALI, we selected 10 miRNAs from the top 10 DE-miRNAs signature and previous bibliography retrieval for RT-qPCR in a validation cohort (20 ATAAD patients with or without ALI, respectively): hsa-miR-181d-5p, hsa-miR-423-5p, hsa-miR-145-5p, hsa-miR-206, hsa-let-7e-5p, hsa-miR-103a-3p, hsa-miR-26b-3p, hsa-miR-485-5p, hsa-miR-29a-3p and hsa-miR-483-3p (Supplementary Table S3 online).

**Figure 6:**
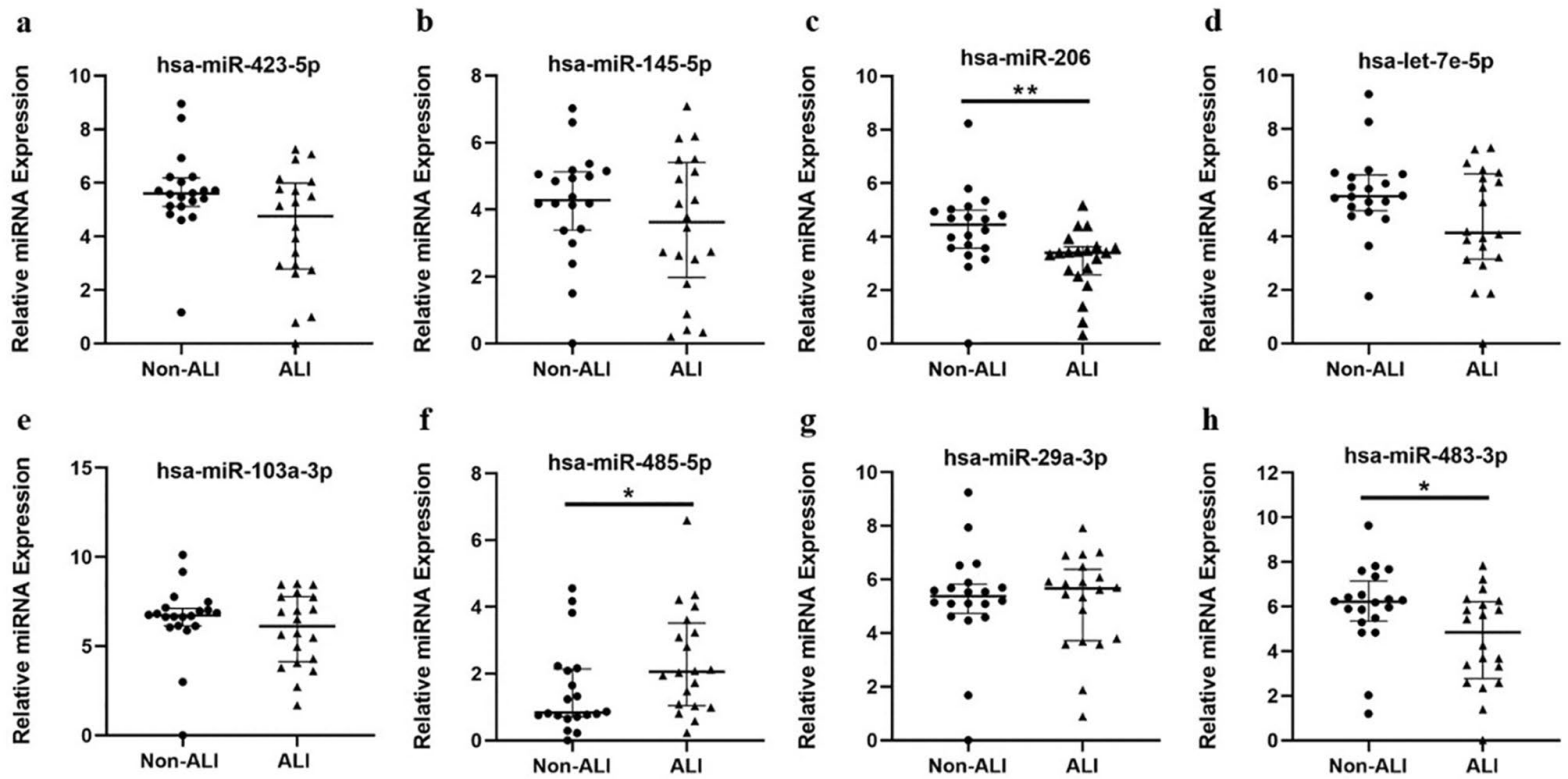
Validation of candidate DE-miRNAs by RT-qPCR in the Non-ALI and ALI groups. (**a–h**) Relative expression levels of selected miRNAs in the Non-ALI and ALI groups were detected by RT-qPCR (n = 20 in each group). The results were normalized to cel-miR-39. Data were analyzed by Mann–Whitney *U* test. Hsa-miR-181d-5p and hsa-miR-26b-3p could not be detected owing to their low expression levels. *DE-miRNAs* differentially expressed miRNAs; *RT-qPCR* real-time quantitative polymerase chain reaction; *Non-ALI group* patients with acute type A aortic dissection without acute lung injury, *ALI group* patients with acute type A aortic dissection with acute lung injury. *P < 0.05; **P < 0.01.

As shown in Fig. 6a–h, of the ten candidate miRNAs, the relative expression levels of 3 miRNAs (hsa-miR-485-5p, hsa-miR-206 and hsa-miR-483-3p) were significantly different between the non-ALI group and the ALI group. The expression of hsa-miR-485-5p (upregulated in ALI group, *P* < 0.05) and hsa-miR-206 (downregulated in ALI group, *P* < 0.01) were consistent with sequencing results. Hsa-miR-181d-5p and hsa-miR-26b-3p could not be detected due to their extremely low expression levels in all exosome samples.

## Discussion

In this study, we analyzed the profile of plasma exosomal miRNAs in ATAAD patients with ALI and selected the top 10 downregulated and upregulated DE-miRNAs in 283 specific miRNAs for further functional analysis. Initially, SMAD2, MYOCD, MKL1, SMAD4, SMAD3, NR3C1, GTF2I, NFIC, SRC, and RREB1 were predicted as the TFs of the downregulated DE-miRNAs and CEBPB, IL4, HOXD10, GLI1, SRSF1, ZMYND8, CCNT2, USF1, MKL1 and HMCA1 were predicted as the TFs of the upregulated DE-miRNAs. Additionally, the biological functions and pathways of predictive target genes of these DE-miRNAs were involved in the regulation of response to hypoxia, NK cell proliferation, NK T cell proliferation, acute inflammatory response, cell junction, cytoskeleton, HIF-1 signaling pathway, NF-κB signaling pathway, necroptosis and adherent junction. Finally, RHOA and INSR were considered hub genes in predictive target genes of DE-miRNAs. For further validation, we found that the expression of hsa-miR-485-5p and hsa-miR-206 showed the same trend with regard to sequencing results, which indicated their regulatory role in the pathogenesis of ATAAD with ALI.

Preoperative ALI is a frequent and severe complication in ATAAD, and hypoxemia is the most typical symptom in these patients. It has been reported that ATAAD patients complicated with ALI have longer the duration of ventilation and ICU and hospital stays, which leads to a poor clinical prognosis^17^. As a result, a better understanding of the pathophysiology of the ATAAD with ALI can improve clinical prophylaxis and treatments in these patients. Exosomes are small membrane vesicles that are rich in miRNAs and can protect miRNAs from the degradation of RNase in circulation, which act as the carriers of intercellular communication^18^. Recent studies revealed that plasma exosomal miRNAs are closely related to the onset of ALI^19,20^. Jiang et al.^20^ explored the influence of circulating exosomes on macrophage activation of sepsis-related ALI and demonstrated that exosomal miR-155 promoted macrophages proliferation and inflammation by targeting SHIP1 and SOCS1. Another study showed that exosome-shuttling miR-1-3p increased PMVECs permeability and membrane injury by targeting SERP1, leading to vascular barrier dysfunction^19^. However, the association between the plasma exosomal miRNAs and ATAAD with ALI has not been reported.

In our present study, we conducted RNA sequencing and identified a total of 283 specific plasma exosomal miRNAs in ATAAD patients with ALI. Based on its pathophysiological process, these miRNAs might be released from a variety of activated inflammatory cells (e.g. neutrophils, lymphocytes, monocytes, etc.), aortic and pulmonary vascular endothelial cells, and even alveolar epithelial cells via exosomes, which were transferred into the circulation^21,22^. In these DE-miRNAs, we eliminated those novel miRNAs or low expression level miRNAs in each sample and finally, selected the top 10 downregulated (hsa-miR-181d-5p, hsa-miR-31-3p, hsa-miR-326, hsa-miR-423-5p, hsa-miR-145-5p, hsa-miR-744-5p, hsa-miR-99b-3p, hsa-miR-214-3p, hsa-miR-206, hsa-let-7e-5p) and upregulated (hsa-miR-103a-3p, hsa-miR-26b-3p, hsa-miR-1306-5p, hsa-miR-7-1-3p, hsa-miR-3120-3p, hsa-miR-335-3p, hsa-miR-485-5p, hsa-miR-664a-3p, hsa-miR-29a-3p, hsa-miR-483-3p) DE-miRNAs for further studies.

By predicting TFs of these DE-miRNAs, we found that SMAD2, MYOCD, MKL1, SMAD4, SMAD3, NR3C1, GTF2I, NFIC, SRC, RREB1 were predicted to be the downregulated DE-miRNAs TF, while CEBPB, IL4, HOXD10, GLI1, SRSF1, ZMYND8, CCNT2, USF1, MKL1 and HMCA1 proteins were predicted to be the upregulated DE-miRNAs TF. Among these TFs, the SMAD proteins family might potentially modulate the expression of the main downregulated DE-miRNAs. Previous studies showed that SMAD proteins could control DROSHA-mediated miRNA maturation in human smooth muscle cells^23^. Recent research expanded the results and indicated that they were involved in miRNA transcription. For instance, Du et al.^24^ demonstrated that SMAD4 regulated miR-425-TGF-β signaling pathway, resulting in granulosa cell apoptosis. Their influence on miRNAs in osteoclast differentiation was also well documented^25^. In the predictive TFs of upregulated DE-miRNAs, SRSF1, an RNA binding protein, had been shown to modulate miRNAs processing such as miR-7, miR-10b, miR-29b^26^. Studies indicated that SRSF1 regulated the expression of miR-7 through binding to the pri-miR-7 sequence and promoting DROSHA cleavage^27^. It can also modulate miR-10b and miR-29b in the same way, which played a critical role in immune response and tumorigenesis^28,29^. Besides, USF1 can activate the expression of miR-132 by binding to the E-box in the miR-132 promoter and influence its synthesis in oxygen and glucose deprivation-induced cell apoptosis^30^. More researches about the role of these predictive TFs in ATAAD with ALI need to be further conducted.

According to GO and KEGG functional analysis, the top 20 enrichments in BP, CC and MF of GO were listed and showed that certain miRNAs participate in biological processes or functions associated with response to hypoxia, natural killer (NK) cell proliferation, NK T cell proliferation, acute inflammatory response, cell junction and cytoskeleton. Numerous evidence has supported that ATAAD with ALI is characterized by widespread uncontrolled inflammation in the lungs, a functional or structural breakdown in pulmonary vascular endothelial junction and barrier damage, resulting in severe oxygenation impairment in body^31^. Hypoxia can be further pro-inflammatory and lead to a breakdown of vascular barriers. But the adaptive response to hypoxia, especially hypoxia-inducible transcription factors (HIF) stabilization and induction of its downstream genes, bears anti-inflammatory and lung tissue-protective aspects, which highlight the functional role of hypoxia signaling in ALI^32^. It has been reported that the expression of many miRNAs changes under hypoxia, and these miRNAs may modulate HIF expression or switch, but the results are controversial^33^. Besides, most of these studies have focused on cancer cell lines only and often ignore endothelial or epithelial cells, so that the exact role of hypoxia-induced miRNAs in them remains unclear^33^. Our results above further, suggested that specific plasma exosomal miRNAs were involved in the pathogenesis of ATAAD with ALI by recruiting immune cells, expanding inflammatory response, destroying cell junction and skeleton (a model figure was shown in Supplementary Fig. S3). In addition, HIF-1 signaling pathway, NF-κB signaling pathway, necroptosis and adherens junction were listed in the top 20 results of KEGG analysis, and these biological pathways were also closely related to ATAAD with ALI^34^. Recent evidence revealed that the activation of the HIF-1 signaling pathway could amplify the inflammatory response by stimulating the release of inflammatory factors, and promote the apoptosis of alveolar type II epithelial (AT-II) cells, which exacerbated the ALI^35^. The NF-κB family controls various processes such as immunity and inflammation^36^. Chen et al.^37^ confirmed that NF-κB signaling pathway was abnormally activated in the AT-II of patients with ALI and promoted the production of inflammatory factors including IL-1β, IL-6 and TNF-α, which initiated the inflammatory cascade. Besides, necroptosis might be involved in endothelial injury^38^ and adherens junction were critical for basal pulmonary microvascular integrity^39^. These results were similar to those of GO analysis, indicating the potential role of selected DE-miRNAs in ATTAD with ALI.

Next, we constructed the PPI network of those predicted target genes and identified the top 10 hub genes. Inspiringly, the changes in the 20 genes expression were generally consistent with previous studies’ results. For the upregulated genes, we found that RAS homolog family member A (RHOA) ranked first. Recent studies showed that RHOA and its downstream effector were closely related to the pathogenesis of ALI^40^. Upregulation of the RHOA/Rho kinase (ROCK) signaling pathway promotes immune cell migration and adhesion, accelerates pulmonary endothelial cells’ apoptosis, and these effects result in endothelium barrier dysregulation and edema—the hallmarks of ALI^26^. Besides, other hub genes such as janus kinase 3 (JAK3)^41^, calmodulin 1 (CALM1) ^42^ and ribosomal protein L13 (RPL13) ^43^ have also been reported to act as risk factors involved in the onset of ALI. For the downregulated genes, insulin receptors (INSR) had the highest connectivity degrees. It binds insulin to produce biological effects and is positively correlated with insulin level in the body^44^. Emerging evidence revealed that hyperinsulinemia had a protective effect on obese rats with lipopolysaccharide (LPS)-induced ALI by increasing their alveolar fluid clearance^45^. Furthermore, peroxisome proliferator-activated receptor-alpha (PPARA), as the predictive downregulated hub gene, has also been reported that it reduces inflammation and vascular leakage in a murine model of ALI^46^. These hub genes mentioned above may play a potential role in exploring novel mechanisms and therapeutic targets in ATAAD with ALI.

Moreover, based on sequencing results and previous bibliography retrieval, we selected 10 candidate miRNAs for validation. The RT-qPCR confirmed that hsa-miR-485-5p was significantly upregulated and hsa-miR-206 was significantly downregulated in ALI group. Of the two specific miRNAs, miR-206 has been previously reported to be associated with ALI. Zhou et al. ^47^ demonstrated that miR-206 expression was significantly decreased in lung tissue of sepsis-induced ALI mouse model, showing the same trend as our findings in plasma exosomes in ALI group, and the phenotype of ALI including the lung tissue inflammatory response, wet to dry weight ratio (W/D) and bronchoalveolar lavage fluid (BALF) could be attenuated by injection of miR-206 agomir. Further in vitro experiments revealed that downregulated miR-206 increased the permeability of alveolar air-blood barrier and promoted the development of ALI by targeting CX43 mRNA and upregulating CX43 expression in AT-II cells. These pathological changes above are also common in ATAAD-induced ALI^4^, suggesting a potential role of miR-206 in its pathogenesis. What’s more, miR-206 was found to be related to inflammation, apoptosis and autophagy^8,48,49^. For miR-485-5p, a relative study with reference to ATAAD or ALI is lacking. Existing evidence showed that it mediated inflammatory factors (TNF-α, IL-6, IL-8, etc.) involved in inflammation^50^. Other studies have reported its association with suppression of cell proliferation and migration^51,52^. Thus, we speculate that miR-485-5p may be involved in ATAAD with ALI by regulating inflammation and promoting pulmonary capillary endothelial damage. However, its precise function in this process remains to be elucidated.

As far as we know, this study is the first to explore the DE-miRNAs in plasma exosomes from ATAAD patients with ALI. We measured the plasma exosomal miRNA profile of these patients, which provided us with a novel source of biomarkers for this disease. In addition, plasma exosomal miRNAs bind and enter their target cells through the circulation to regulate the post-transcriptional process. Such mediation can help us better clarify the underlying mechanisms of ATAAD with ALI. However, there are still several limitations. Firstly, the validation cohort in this study was relatively small. The results need to be verified in a larger population. Secondly, the present study was lack of in vitro and in vivo experimental validation of the functions of the two confirmed exosomal miRNAs in ATAAD with ALI. Corresponding experiments will be performed in the future to gain insight into its pathogenesis.

## Conclusion

In summary, this study has identified differential expression profile of plasma exosomal miRNAs in ATAAD with ALI, which were related to inflammation and dysfunction of cells junction. Further research is required to explore their potential role in pathogenic mechanisms.

## Methods

### Patients and ethics

59 patients diagnosed with ATAAD confirmed by computed tomographic angiography (CTA) of aorta according to 2014 ESC Guidelines^53^ were recruited at Xiangya Hospital of Central South University, from July to December in 2020. 19 Patients were enrolled as the discovery cohort (Non-ALI group, n = 8; ALI group, n = 11) and 40 patients were enrolled as the validation cohort (Non-ALI group, n = 20; ALI group, n = 20). Plasma exosome miRNAs were evaluated by next-generation sequencing (NGS) in the discovery cohort, and then, selected miRNA candidates from the differential expression profile of plasma exosomal miRNAs were validated by RT-qPCR in the validation cohort.

The diagnosis of ALI was based on the American-European Consensus Conference (AECC) definition^54^. The following four criteria were required to be present: (1) acute onset; (2) P_aO2_/F_iO2_ ≤ 300 mmHg [regardless of positive end-expiratory pressure (PEEP) level]; (3) bilateral infiltrates seen on frontal chest radiograph; (4) pulmonary artery wedge pressure (PAWP) ≤ 18 mmHg when measured or no clinical evidence of left atrial hypertension. Patients with the following conditions were excluded: those with chronic aortic dissection, chronic lung diseases, chronic renal or liver failure, immune system diseases, connective tissue diseases or malignant diseases.

The present study was approved by the Medical Ethical Committee of the Xiangya Hospital of Central South University (Hunan, China) and conducted following their guidelines and regulations. All patients provided written informed consent.

### Exosome isolation

Whole blood (5–10 ml) was sampled from each patient on admission, centrifuged at 3000 rpm for 10 min at 4 °C. The collected plasma samples were stored − 80 °C until required. Exosomes were extracted by differential ultra-centrifugation as follows. Briefly, plasma (4 ml) was centrifuged at 12,000 rpm for 10 min at 4 °C to remove cell debris. The supernatant fluid was diluted with 20 ml phosphate-buffered saline (PBS) and filtered through a 0.22 µm disposable filter. Then, it was transferred to a ultracentrifuge (Beckman Coulter, Inc., Brea, CA) and ultracentrifuged using a Type 70Ti rotor (Beckman Coulter, Inc., Brea, CA) at 120,000×*g* for 90 min at 4 °C twice. The pellets containing exosomes were suspended in 200 µl PBS for further application.

### TEM

For electron microscopy analysis, isolated exosomes suspended in PBS were dropped on copper-coated grids and stained with 2% phosphotungstic acid. After the grids were completely dried at room temperature, they were visualized using a Hitachi 7800 TEM (H-7800, Hitachi Ltd., Tokyo, Japan).

### NTA

NTA were performed using a NanoFCM N30E instrument (N30E, Fujian, China) according to the manufacturer’s instruction. The exosome samples were diluted in PBS. The NanoFCM instrument captured the Brownian motion of the extracted nanoparticles and measured their size and concentration.

### Western blot

The isolated exosomes were lysed in RIPA lysis buffer containing a protease inhibitor to extract protein and the samples were quantified by a BCA protein assay kit. Equal amounts of protein were separated on a 10% or 15% sodium dodecyl sulfate (SDS)—polyacrylamide gel electrophoresis (PAGE), and the bands were transferred to polyvinylidene fluoride (PVGF) membranes. After blocking with 5% skimmed milk for 1 h, the membranes were incubated primary antibodies at 4 °C overnight and then incubated with peroxidase-conjugated anti-rabbit or anti-mouse secondary antibodies for 1 h at room temperature. Finally, the membranes were visualized and analyzed using the ChemiDoc MP Imaging system (Bio-Rad, Hercules, CA, USA).

The primary antibodies included anti-tumor susceptibility gene 101 protein (TSG 101) antibody (ab125011, 1:1000, Abcam), anti-CD63 antibody (A5271, 1:1000, Abclonal) and anti-Calnexin antibody (ab22595, 1:1000, Abcam).

### Exosomal RNAs extraction and NGS

Total RNA was extracted from plasma exosomes samples using miRNeasy^®^ Mini kit (Qiagen, Cat. No. 217004). The RNA quality and quantity were evaluated by the RNA Nano 6000 Assay Kit of the Agilent Bioanalyzer 2100 System (Agilent Technologies, CA, USA). Then, the samples were used to establish a sequencing library and the quality of the library was tested with an Agilent 2100 bioanalyzer before the NGS.

Small RNA sequencing (16-30nt) was performed using the BGISEQ-500 sequencer (BGI Park, Shenzhen, China). Low-quality data were removed, and a substantial amount of small RNAs, which mapped perfectly to the human genome (ftp://ncbi.nlm.nih.gov/genomes/Homo_sapiens) were obtained for further analysis. The miRNA expression levels were estimated by the value of fragments perkiobase million (FPKM) and their relative expression levels were assessed by the DEGseq method. Quantitative data for each miRNA from the ALI and non-ALI group was compared to obtain absolute fold change (FC) values, and then log_2_ FC transformation were performed. Differentially expressed miRNAs (DE-miRNAs) was defined by an absolute value of log_2_ FC > 0.8, the *Q*-value (adjusted *P*-value) < 0.05 and the value of FPKM in each sample > 0.

### Prediction of miRNAs potential TFs

The upstream TFs of DE-miRNAs were predicted using FunRich software^55^. The selected up-regulated or down-regulated miRNAs were inputted into the software, and the results showed the top 10 predictive TFs.

### Prediction of miRNAs target genes and functional and pathway analysis

The downstream target genes of DE-miRNAs were predicted using three online database: TargetScan (http://www.targetscan.org/vert_72/) ^56^, miRbase (https://www.mirbase.org/) ^57^ and RNAhybrid (https://bibiserv.cebitec.uni-bielefeld.de/rnahybrid/) ^58^. The classification of main function annotation of predictive target genes was performed by GO analysis (www.geneontology.org) ^59^ and the identification of molecular pathways was applied by KEGG analysis (www.genome.jp/kegg) ^60^.

### PPI network construction and analysis

The predicted target genes of the top 10 down- and upregulated DE-miRNAs were submitted to the STRING database which was used for a PPI network construction (http://string-db.org) ^61^. A composite score > 0.4 was defined as a significant interaction. Subsequently, the results were loaded into Cytoscape for visualization, and the top 10 hub genes were calculated by degree scoring^62^.

### RT-qPCR of DE-miRNAs

Total RNA was reverse transcribed by using PrimeScript™ RT reagent Kit (Perfect Real Time) (Takara, RR037A). The miRNA expression was examined by RT-qPCR using TaqMan^®^ probe, and the sequence of primers and probes were shown in Supplementary Table S4 online. Each sample was examined in triplicate. The relative expression levels of exosomal miRNAs was analyzed by the ΔΔCt method relative to cel-miR-39.

### Statistical analysis

Shapiro–Wilk tests were applied to test if the continuous variables conform to the normal distribution. Normally distributed continuous variables were presented as the mean ± SD while non-normally distributed continuous variables were presented as the median and interquartile range (IQR). Categorical variables were presented by number and percentage. Continuous data were compared using the Student *t* test or Mann–Whitney *U* test depending on the data’s distribution, and categorical data were compared using the chi-squared test. A two-tailed *P* value < 0.05 was considered statistically significant. Statistical analyses were performed using SPSS 25.0 software (SPSS, Inc., Chicago, IL, USA).

## Supplementary Information

Supplementary Tables.

Supplementary Legends.

Supplementary Figure S1.

Supplementary Figure S2.

Supplementary Figure S3.

Supplementary Figure S4.

Supplementary Figure S5.

Supplementary Figure S6.

## Data Availability

The data analyzed in this study are available from the corresponding author on reasonable request.

## Abbreviations

ATAAD: Acute type A aortic dissection
ALI: Acute lung injury
ICU: Intensive care unit
miRNAs: MicroRNAs
MHC: Myosin heavy chain
RhoB: Ras homolog gene family member B
IBD: Inflammatory bowel disease
IL-6: Interleukin-6
CTA: Computed tomographic angiography
RT-qPCR: Real-time quantitative polymerase chain reaction
AECC: American-European Consensus Conference
P_aO2_: Arterial oxygen tension
F_iO2_: Inspiratory oxygen fraction
PEEP: Positive end-expiratory pressure
PAWP: Pulmonary artery wedge pressure
PBS: Phosphate buffered saline
TEM: Transmission electron microscopy
NTA: Nanoparticle tracking analysis
SDS: Sodium dodecyl sulfate
PAGE: Polyacrylamide gel electrophoresis
PVGF: Polyvinylidene fluoride
TSG 101: Anti-tumor susceptibility gene 101 protein
NGS: Next-generation sequencing
FPKM: Fragments perkiobase million
FC: Fold change
DE-miRNAs: Differentially expressed miRNAs
TFs: Transcription factors
GO: Gene Ontology
KEGG: Kyoto Encyclopedia Genes and Genomes
PPI: Protein–protein interaction
3′-UTR: 3′-Untranslated region
LVEF: Left ventricular ejection fraction
BP: Biological process
CC: Cellular component
MF: Molecular functions
RHOA: Ras homolog family member A
ROCK: Rho kinase
JAK3: Janus kinase 3
CALM1: Calmodulin 1
RPL13: Ribosomal protein L13
INSR: Insulin receptor
PPARA: Peroxisome proliferator-activated receptor-alpha
W/D: Wet to dry weight ratio
BALF: Bronchoalveolar lavage fluid
AT-II: Alveolar type II epithelial cells
