# Supplementary material for: Differential expression profile of plasma exosomal microRNAs in acute type A aortic dissection with acute lung injury
Source: Sci Rep. 2022 Jul 8;12:11667. doi: 10.1038/s41598-022-15859-3 (PMC9270349; doi:10.1038/s41598-022-15859-3)
Supplement: Supplementary file 1 — Supplementary Tables. [file 41598_2022_15859_MOESM1_ESM.doc]

**Differential expression profile of plasma exosomal microRNAs in acute type A aortic dissection with acute lung injury**

Chiyuan Zhang, MD^1^; Hui Bai, MD^2^; Lei Zhang, MD^2^; Yanfeng Zhang, MD^2^; Xuliang Chen, MD^2^; Ruizheng Shi, PhD^3^; Guogang Zhang, PhD^1^; Qian Xu, MD^2*^; Guoqiang Lin, PhD^2*^

**Tables**

|  | **Non-ALI group (n=8)** | **ALI group (n=11)** | ***P* value** |
| --- | --- | --- | --- |
| Age (years) | 56.8±9.6 | 48.6±8.1 | 0.059 |
| Sex，male (%) | 6 (75.0) | 6 (54.5) | 0.633 |
| BMI (kg/m^2^) | 22.5±2.9 | 25.3±2.2 | 0.133 |
| Respiratory rate (/min) | 20.0 (19.0-20.0) | 20.0 (20.0-25.0) | 0.008 |
| Heart rate (/min) | 68.4±5.6 | 84.3±11.5 | 0.002 |
| SBP (mmHg) | 154.8±29.5 | 158.6±35.4 | 0.808 |
| DBP (mmHg) | 73.3±19.6 | 86.18±20.9 | 0.190 |
| Sepsis (%) | 0 (0.0) | 0 (0.0) | 1.000 |
| Pneumonia (%) | 6 (75.0) | 9 (81.8) | 1.000 |
| Trauma (%) | 0 (0.0) | 0 (0.0) | 1.000 |
| Hypertension (%) | 4 (50.0) | 9 (81.8) | 0.319 |
| Diabetes (%) | 0 (0.0) | 0 (0.0) | 1.000 |
| CAD (%) | 0 (0.0) | 0 (0.0) | 1.000 |
| Hyperlipemia (%) | 1 (12.5) | 1 (9.1) | 1.000 |
| Smoking (%) | 6 (75.0) | 4 (36.4) | 0.170 |
| Drinking (%) | 1 (12.5) | 1 (9.1) | 1.000 |
| WBC (×10^9^/L) | 12.2±2.9 | 15.3±4.8 | 0.124 |
| NT-proBNP (pg/ml) | 230.5 (124.5-438.5) | 122.5 (100.0-369.1) | 0.321 |
| P_aCO2_ (mmHg) | 42.4±5.3 | 41.4±6.0 | 0.709 |
| P_aO2_ (mmHg) | 186.6±49.3 | 90.5±22.6 | 0.001 |
| F_iO2_ (%) | 37.0 (37.0-40.0) | 38.0 (37.0-41.0) | 0.414 |
| P_aO2_/ F_iO2_ | 492.5±98.8 | 227.4±53.1 | <0.001 |
| LVEF (%) | 60.4±4.4 | 65.0±4.5 | 0.039 |

**Supplementary Table S1.** Clinical and demographic characteristic of patients in discovery cohort. Data are presented as mean ± SD, n(%), or medians(interquartile ranges). Non-ALI group: patients with acute type A aortic dissection without acute lung injury; ALI group: patients with acute type A aortic dissection with acute lung injury; BMI: body mass index; SBP: systolic blood pressure; DBP: diastolic blood pressure; CAD: coronary artery disease; P_aCO2_: partial pressure of carbon dioxide in artery; P_aO2_: arterial oxygen tension; F_iO2_: inspiratory oxygen fraction; LVEF: left ventricular ejection fraction.

|  | **Non-ALI group (n=20)** | **ALI group (n=20)** | ***P* values** |
| --- | --- | --- | --- |
| Age (years) | 56.6±13.9 | 51.2±10.4 | 0.172 |
| Sex，male (%) | 12 (60.0) | 14 (70.0) | 0.507 |
| BMI (kg/m^2^) | 23.2±3.2 | 25.3±2.1 | 0.065 |
| Respiratory rate (/min) | 20.0 (18.0-20.0) | 20 (19.0-21.8) | 0.217 |
| Heart rate (/min) | 80.4±12.9 | 88.7±20.2 | 0.127 |
| SBP (mmHg) | 160.2±35.4 | 158.6±25.0 | 0.870 |
| DBP (mmHg) | 77.9±21.1 | 84.8±16.7 | 0.255 |
| Sepsis (%) | 0 (0.0) | 0 (0.0) | 1.000 |
| Pneumonia (%) | 7 (35.0) | 12 (60.0) | 0.113 |
| Trauma (%) | 0 (0.0) | 0 (0.0) | 1.000 |
| Hypertension (%) | 14 (70.0) | 20 (100.0) | 0.02 |
| Diabetes (%) | 0 (0.0) | 0 (0.0) | 1.000 |
| CAD (%) | 1 (5.0) | 0 (0.0) | 1.000 |
| Hyperlipemia (%) | 2 (10.0) | 0 (0.0) | 0.487 |
| Smoking (%) | 12 (60.0) | 14 (70.0) | 0.507 |
| Drinking (%) | 2 (0.0) | 2 (10.0) | 1.000 |
| WBC (×10^9^/L) | 9.5 (7.9-14.5) | 13.45 (11.1-15.0) | 0.062 |
| NT-proBNP (pg/ml) | 202.2 (100.0-459.8) | 184.2 (100.0-302.9) | 0.424 |
| P_aCO2_ (mmHg) | 40.0 (36.5-43.75) | 41.5 (37.3-45.5) | 0.664 |
| P_aO2_ (mmHg) | 178.0 (143.5-204.8) | 92.0 (72.8-99.0) | <0.001 |
| F_iO2_ (%) | 36.5 (30.8-37.8) | 37.0 (35.0-41.0) | 0.116 |
| P_aO2_/ F_iO2_ | 491.0 (418.0-611.8) | 256.5 (203.8-283.0) | <0.001 |
| LVEF (%) | 58.5 (56.3-66.8) | 59.0 (56.0-67.0) | 0.989 |

**Supplementary Table S2.** Clinical and demographic characteristic of patients in validation cohort Data are presented as mean ± SD, n(%), or medians(interquartile ranges). Non-ALI group: patients with acute type A aortic dissection without acute lung injury; ALI group: patients with acute type A aortic dissection with acute lung injury; BMI: body mass index; SBP: systolic blood pressure; DBP: diastolic blood pressure; CAD: coronary artery disease; P_aCO2_: partial pressure of carbon dioxide in artery; P_aO2_: arterial oxygen tension; F_iO2_: inspiratory oxygen fraction; LVEF: left ventricular ejection fraction.

| **Upregulated miRNAs** | | **Downregulated miRNAs** | |
| --- | --- | --- | --- |
| **miRNA** | **log_2_FC** | **miRNA** | **log_2_FC** |
| hsa-miR-103a-3p | 3.45 | hsa-miR-181d-5p | -2.74 |
| hsa-miR-26b-3p | 2.29 | hsa-miR-423-5p | -2.06 |
| hsa-miR-485-5p | 1.21 | hsa-miR-145-5p | -2.05 |
| hsa-miR-29a-3p | 1.17 | hsa-miR-206 | -1.58 |
| hsa-miR-483-3p | 0.97 | hsa-let-7e-5p | -0.97 |

**Supplementary Table S3.** The candidate DE-miRNAs for validation. DE-miRNAs: differentially expressed miRNAs; FC: fold change.

| **miRNA** | **Primer** | **Primer sequence** |
| --- | --- | --- |
| Cel-miR-39 | RT | GTCGTATCCAGTGCAGGGTCCGAGGTATTCGCACTGGATACGACCAAGCT |
|  | F | CGCTCACCGGGTGTAAATC |
|  | P | ATTCGCACTGGATACGACCAAGCT |
| hsa-miR-485-5p | RT | GTCGTATCCAGTGCAGGGTCCGAGGTATTCGCACTGGATACGACGAATTCAT |
|  | F | AATAAGAGGCTGGCCGTG |
|  | P | TTCGCACTGGATACGACGAATTCAT |
| hsa-let-7e-5p | RT | GTCGTATCCAGTGCAGGGTCCGAGGTATTCGCACTGGATACGACAACTATAC |
|  | F | GGCGTGAGGTAGGAGGTT |
|  | P | TTCGCACTGGATACGACAACTATAC |
| has-miR-206 | RT | GTCGTATCCAGTGCAGGGTCCGAGGTATTCGCACTGGATACGACCCACAC |
|  | F | AGGCGTGGAATGTAAGGAAG |
|  | P | TTCGCACTGGATACGACCCACAC |
| has-miR-26b-3p | RT | CGCTTCACGAATTTGCGTGTCAT |
|  | F | GGCCTGTTCTCCATTACTTGG |
|  | P | TTCGCACTGGATACGACAGTACAT |
| has-miR-103a-3p | RT | GTCGTATCCAGTGCAGGGTCCGAGGTATTCGCACTGGATACGACTCATAGCC |
|  | F | ACGCAGCAGCATTGTACAG |
|  | P | TTCGCACTGGATACGACTCATAGCC |
| has-miR-29a-3p | RT | GTCGTATCCAGTGCAGGGTCCGAGGTATTCGCACTGGATACGACTAACCG |
|  | F | CCCGTAGCACCATCTGAAAT |
|  | P | TTCGCACTGGATACGACTAACCGA |
| has-miR-483-3p | RT | GTCGTATCCAGTGCAGGGTCCGAGGTATTCGCACTGGATACGACAAGACG |
|  | F | CGCTCACTCCTCTCCTCC |
|  | P | TCGCACTGGATACGACAAGACGG |
| has-miR-181d-5p | RT | GTCGTATCCAGTGCAGGGTCCGAGGTATTCGCACTGGATACGACACCCAC |
|  | F | AGCCGCCAACATTCATTGT |
|  | P | TTCGCACTGGATACGACACCCAC |
| has-miR-423-5p | RT | GTCGTATCCAGTGCAGGGTCCGAGGTATTCGCACTGGATACGACAAAGTCTC |
|  | F | ACTGAGGGGCAGAGAGC |
|  | P | TTCGCACTGGATACGACAAAGTCTC |
| has-miR-145-5p | RT | GTCGTATCCAGTGCAGGGTCCGAGGTATTCGCACTGGATACGACAGGGAT |
|  | F | CGGTCCAGTTTTCCCAGGA |
|  | P | TCGCACTGGATACGACAGGGA |

**Supplementary Table S4.** The primer sequences of miRNAs for validation experiments. RT: RT primer; F: forward primer; P: probe primer.
