# Supplementary material for: Differential expression profile of plasma exosomal microRNAs in acute type A aortic dissection with acute lung injury
Source: Sci Rep. 2022 Jul 8;12:11667. doi: 10.1038/s41598-022-15859-3 (PMC9270349; doi:10.1038/s41598-022-15859-3)
Supplement: Supplementary file 2 — Supplementary Legends. [file 41598_2022_15859_MOESM2_ESM.doc]

**Differential expression profile of plasma exosomal microRNAs in acute type A aortic dissection with acute lung injury**

Chiyuan Zhang, MD^1^; Hui Bai, MD^2^; Lei Zhang, MD^2^; Yanfeng Zhang, MD^2^; Xuliang Chen, MD^2^; Ruizheng Shi, PhD^3^; Guogang Zhang, PhD^1^; Qian Xu, MD^2*^; Guoqiang Lin, PhD^2*^

**Figure legends**

**Supplementary Figure S1.** Identification of plasma exosomes. (a) TEM showed the exosomes (as indicated by the arrows) were spherical structures with a similar diameter to the exosomes. Scale bar: 100nm. (b) NTA indicated that the diameter distribution of isolated plasma exosomes was consistent with that of exosomes. (c) Representative Western blot revealed that the isolated plasma exosomes were positive for enriched exosome markers CD63 and TSG101, and negative for an intracellular compartment marker Calnexin. TEM: Transmission electron microscopy; NTA: Nanoparticle tracking analysis.

**Supplementary Figure S2.** Volcano plot of DE-miRNAs in the Non-ALI group vs ALI group. Green spots represent significantly downregulated miRNAs, red spots represent significantly upregulated miRNAs and grey spots represent unchanged miRNAs. DE-miRNAs: differentially expressed miRNAs. Non-ALI group: patients with acute type A aortic dissection without acute lung injury; ALI group: patients with acute type A aortic dissection with acute lung injury.

**Supplementary Figure S3.** A model figure to describe the possible mechanism of the impact of plasma exosomal miRNAs on ATAAD with ALI. ATAAD: acute type A aortic dissection; ALI: acute lung injury; 3’-UTR: 3’-untranslated region.

**Supplementary Figure S4.** Western blot for exosome negative markers Calnexin with a full membrane, and its antibody (ab22595, 1:1000, Abcam) was previously characterized^1^.

**Supplementary Figure S5.** Western blot for exosome positive markers CD63 with a full membrane, and its antibody (A5271, 1:1000, Abclonal) was previously characterized^2^.

**Supplementary Figure S6.** Western blot for exosome positive markers TSG101 with a full membrane, and its antibody (ab125011, 1:1000, Abcam) was previously characterized^3^.

**References**

1. Abedi, F., Hayes, A. W., Reiter, R., Karimi, G. Acute lung injury: The therapeutic role of Rho kinase inhibitors. *Pharmacological research*.155**.**104736.(2020).

2. Zhang, W. *et al.* GDF-15 in tumor-derived exosomes promotes muscle atrophy via Bcl-2/caspase-3 pathway. *Cell death discovery*.8**.**162.(2022).

3. Wu, J. Y., Li, Y. J., Hu, X. B., Huang, S., Xiang, D. X. Preservation of small extracellular vesicles for functional analysis and therapeutic applications: a comparative evaluation of storage conditions. *Drug delivery*.28.162-70.(2021).
