# Supplementary figures and images for: Differential expression profile of plasma exosomal microRNAs in acute type A aortic dissection with acute lung injury
Source: Sci Rep. 2022 Jul 8;12:11667. doi: 10.1038/s41598-022-15859-3 (PMC9270349; doi:10.1038/s41598-022-15859-3)

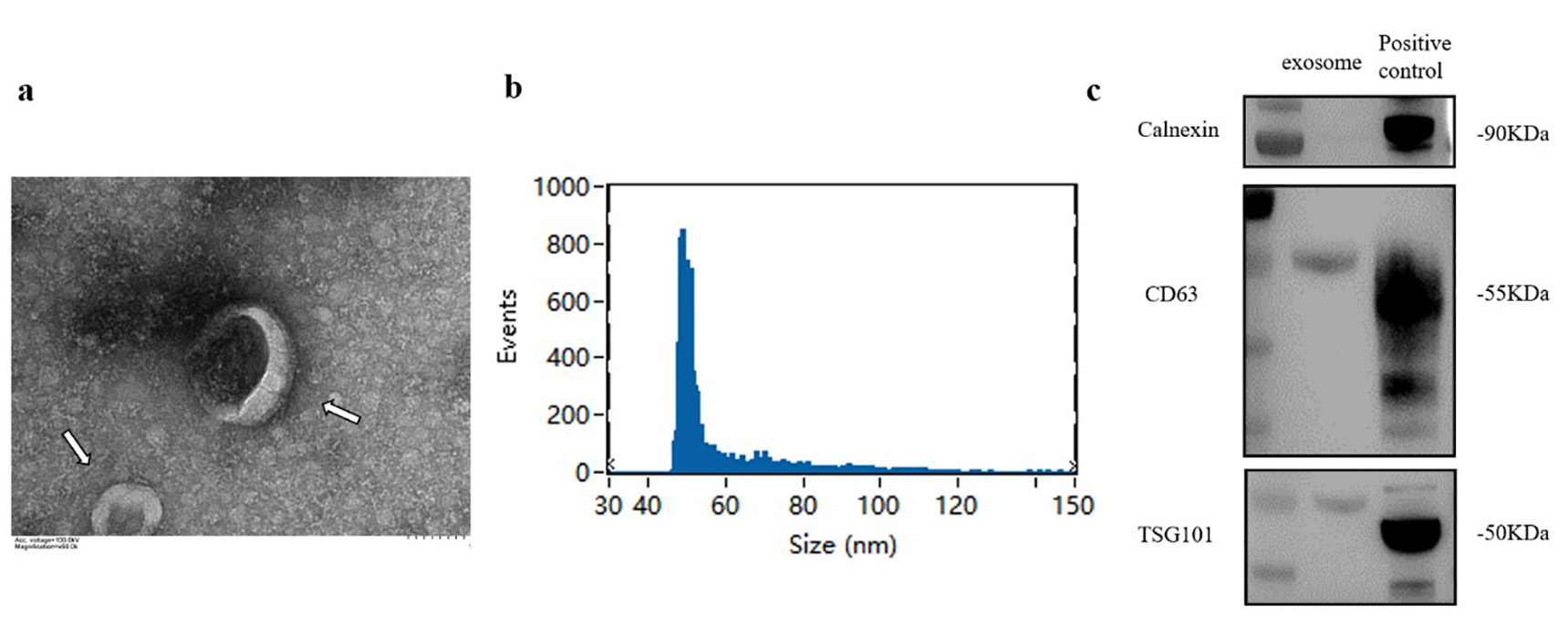

Supplement: Supplementary file 3 — Supplementary Figure S1. [file 41598_2022_15859_MOESM3_ESM.jpg]

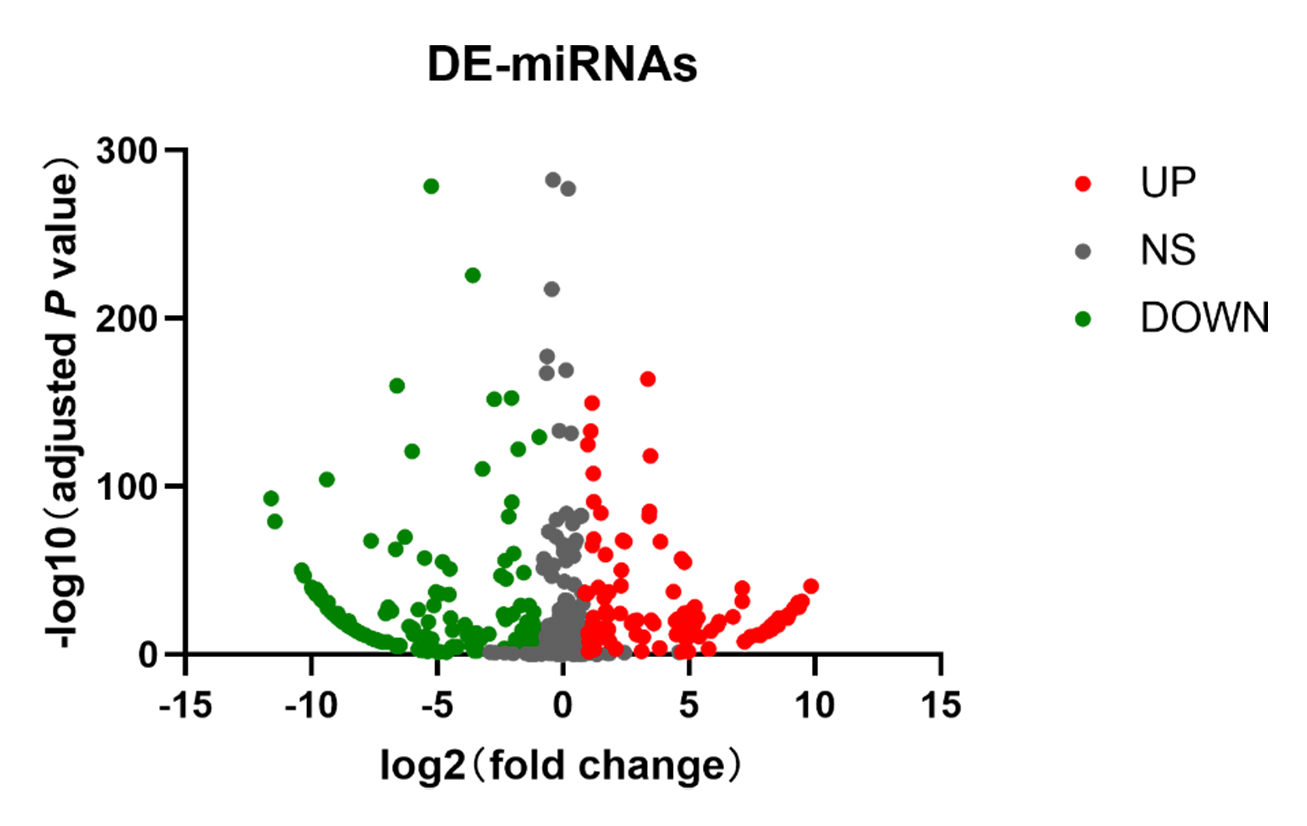

Supplement: Supplementary file 4 — Supplementary Figure S2. [file 41598_2022_15859_MOESM4_ESM.png]

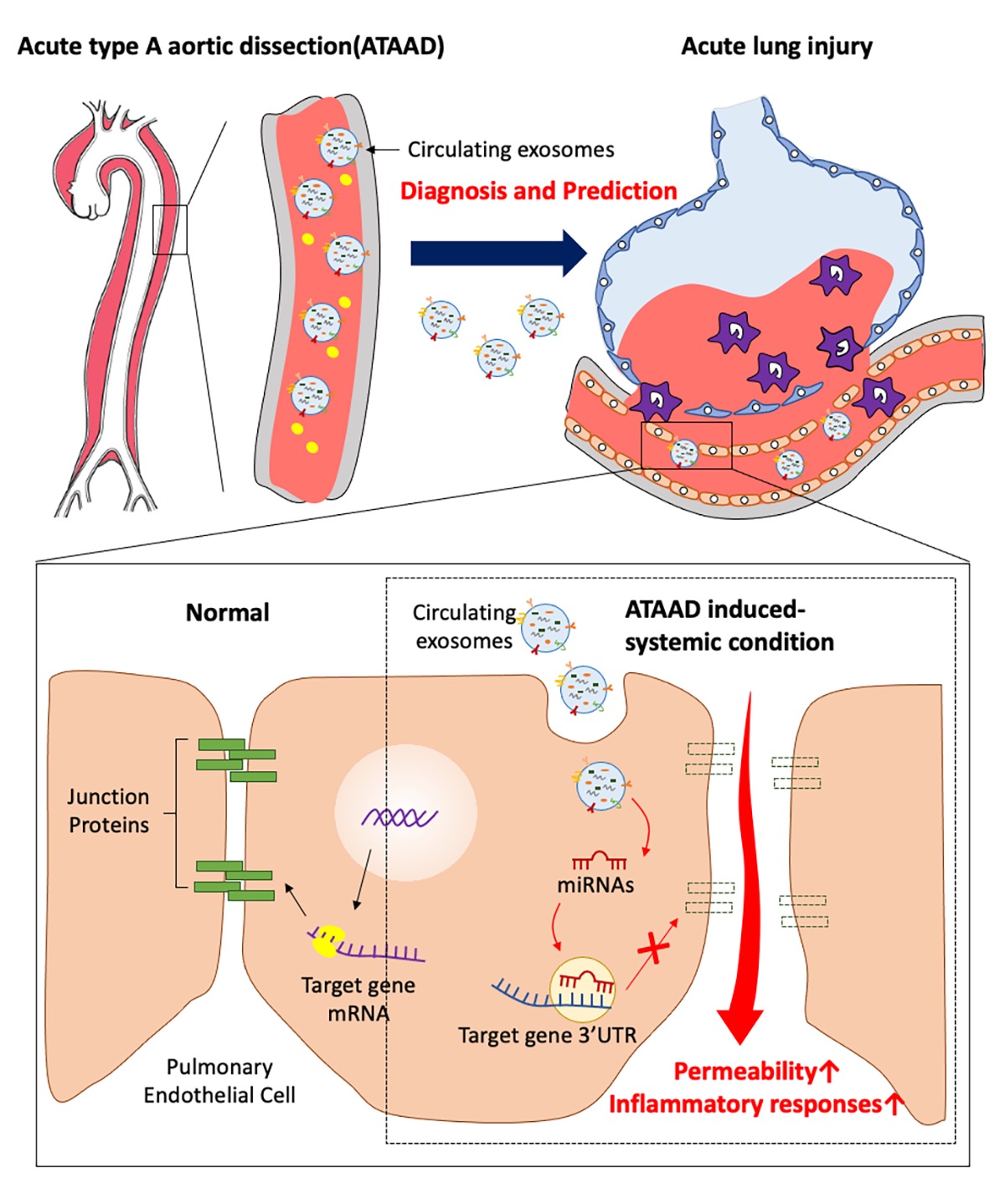

Supplement: Supplementary file 5 — Supplementary Figure S3. [file 41598_2022_15859_MOESM5_ESM.jpg]

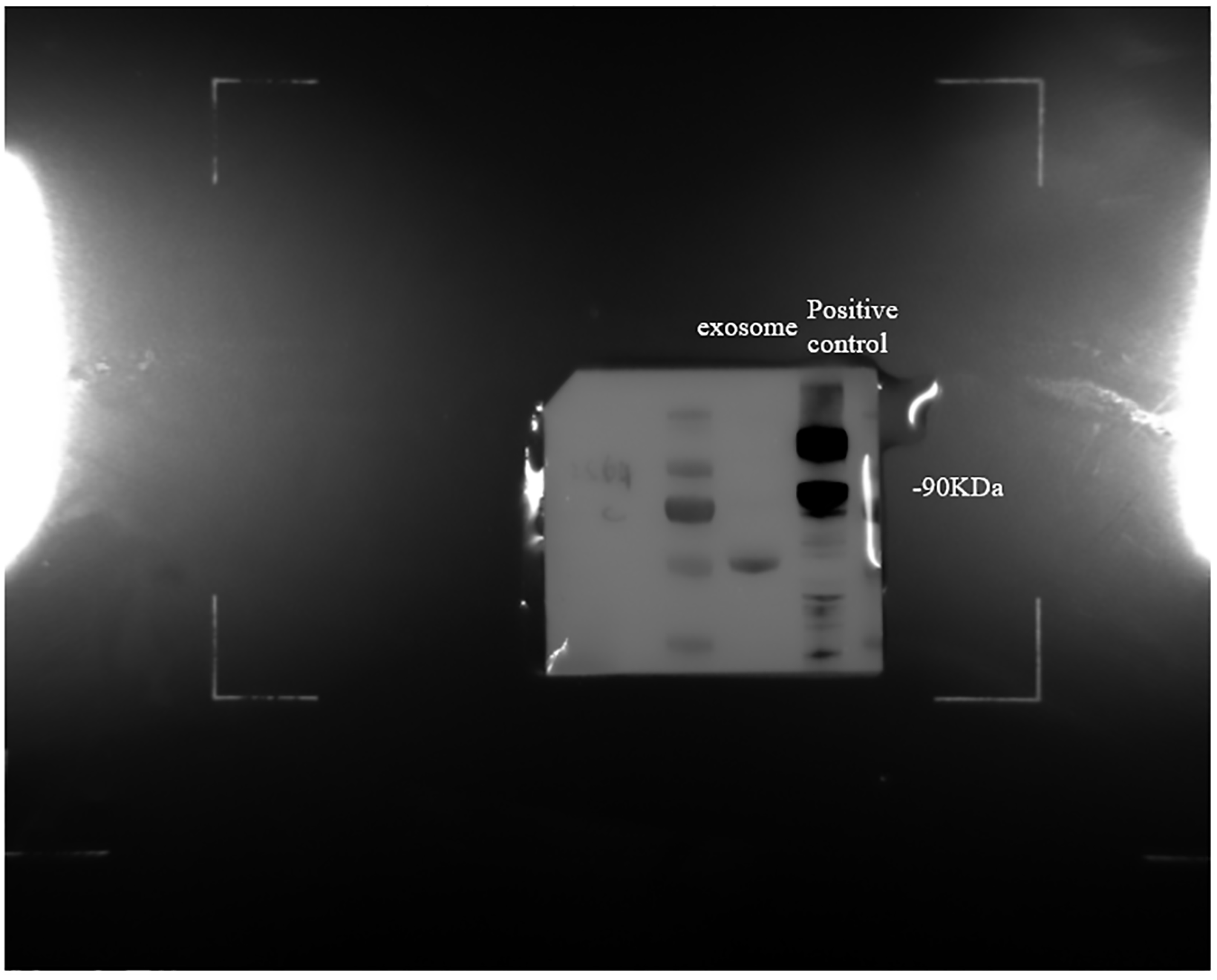

Supplement: Supplementary file 6 — Supplementary Figure S4. [file 41598_2022_15859_MOESM6_ESM.png]

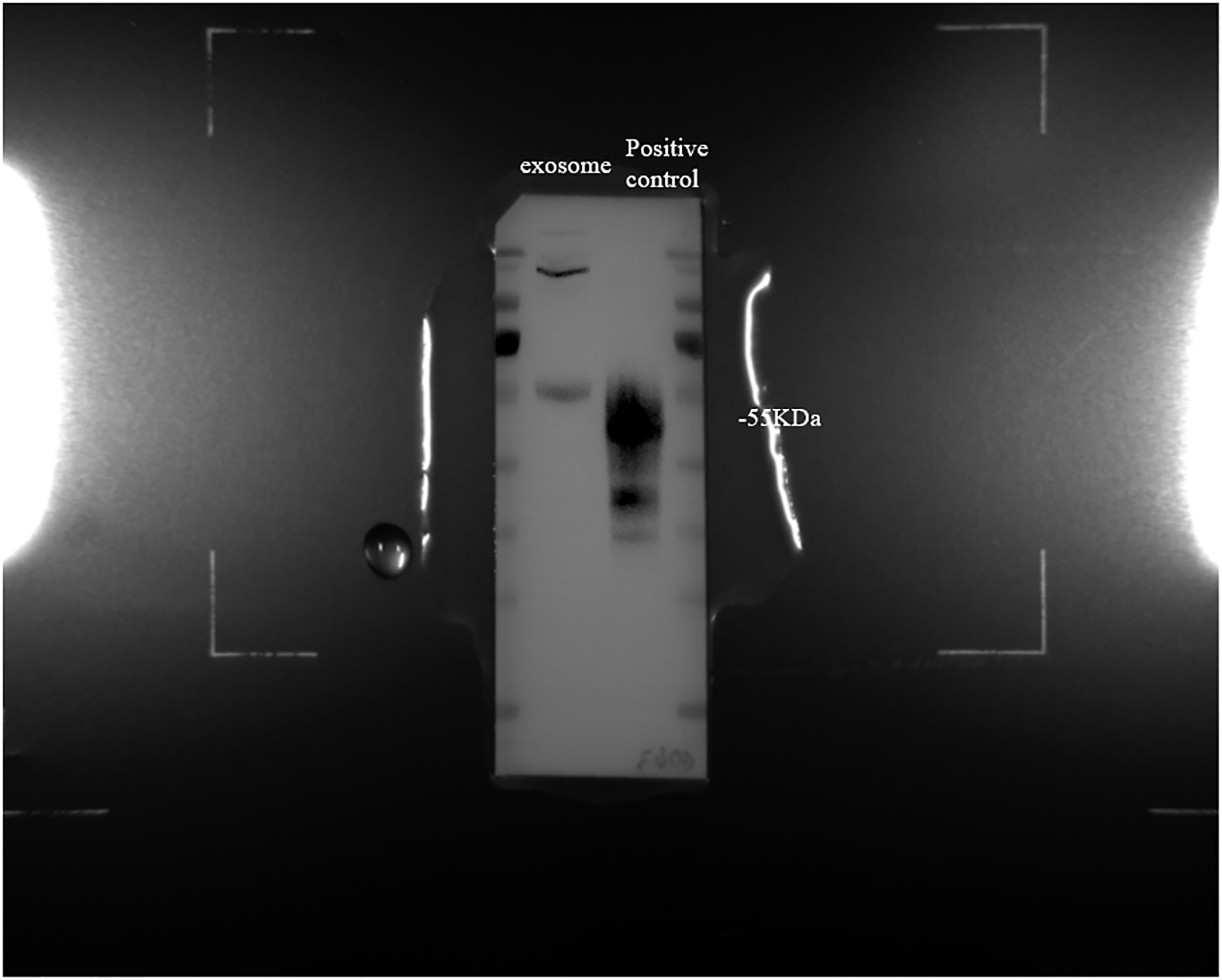

Supplement: Supplementary file 7 — Supplementary Figure S5. [file 41598_2022_15859_MOESM7_ESM.png]

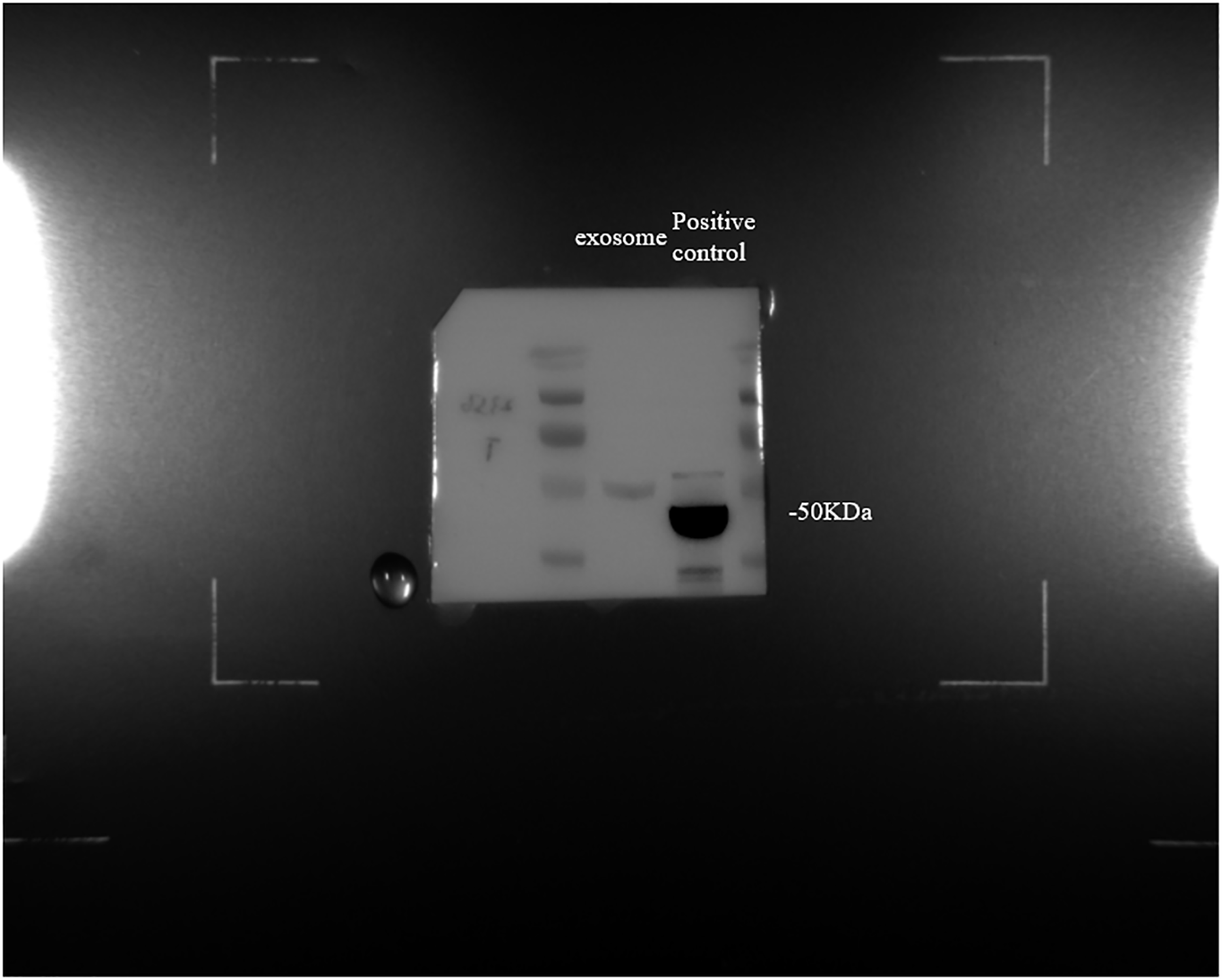

Supplement: Supplementary file 8 — Supplementary Figure S6. [file 41598_2022_15859_MOESM8_ESM.png]
